# Analysis of blood coagulation indexes, thromboelastogram and autoantibodies in patients with recurrent pregnancy loss

**DOI:** 10.12669/pjms.38.7.6284

**Published:** 2022

**Authors:** Huizhen Yao, Yimei Ji, Yanru Zhou

**Affiliations:** 1 Huizhen Yao, Department of Obstetrics, Quzhou People’s Hospital, Quzhou 324000, Zhejiang Province, P.R. China; 2 Yimei Ji, Department of Obstetrics, Quzhou People’s Hospital, Quzhou 324000, Zhejiang Province, P.R. China; 3 Yanru Zhou, Department of Obstetrics, Quzhou People’s Hospital, Quzhou 324000, Zhejiang Province, P.R. China

**Keywords:** Anticardiolipin antibody, Antithyroid antibody, Coagulation index, Recurrent pregnancy loss, Thromboelastogram

## Abstract

**Objectives:**

Changes in coagulation indexes, thromboelastogram(TEG) and autoantibodies in patients with recurrent pregnancy loss (RPL) with different number of abortions were analyzed.

**Methods:**

Medical records of 48 patients with recurrent abortion, treated in Quzhou people’s Hospital from November 2019 to October 2020, were collected as the observation group. Based on the number of abortions, patients were divided into Group-A (Two abortions, n=21), Group-B (Three abortions, n=16) and group C (Abortion ≥ four times, n=11). Records of 50 healthy pregnant women in our hospital in the same period were selected as the control group. Coagulation indexes [prothrombin time (PT), activated partial prothrombin time (APTT), fibrinogen (FIB), D-Dimer (DD)], thromboelastogram (TEG) parameters [reaction time (R), coagulation time(K), maximum thrombus amplitude (MA), coagulation angle (α)], changes in the levels of autoantibodies [anticardiolipin antibody (ACA), anti-endometrial antibody (EmAb), anti-thyroid antibody(ATA)] were compared between the groups.

**Results:**

There were significant differences in the levels of ATPP, Pt, FIB and DD among the groups. Higher number of abortions correlated with lower the levels of ATPP and Pt, and higher levels of FIB and DD (*P<*0.05). Compared to the control group, R and K in Group-A,B and C decreased, while α and MA increased (*P*<0.05). There were significant differences in α and MA indexes. The positive rates of ACA, EmAb and ATA in Group-A were higher than those in the control group, but the difference was not statistically significant (*P*>0.05), while the above indexes in groups B and C were significantly higher than those in the control group (*P*<0.05). The positive rates of ACA and ATA in group C were significantly higher than those in Group-A (*P*<0.05), but there was no significant difference in the positive rate of EmAb (*P*>0.05).

**Conclusion:**

RPL was related to the decrease of APTT, PT, and the increase of FIB and DD levels. TEG indicated that the increase of α and MA values indicated that the risk of multiple abortion was increased. The positive rates of ACA, EmAb and ATA were closely related to multiple abortions, especially the positive rates of ACA and ATA.

## INTRODUCTION

Recurrent pregnancy loss (RPL) is a spontaneous abortion occurring more than two times and two times with the same partner. It is a common complication of obstetric pregnancy with the incidence of about 5% in women of childbearing age.^1^ Some studies have pointed out that the risk of recurrent spontaneous abortion after two consecutive abortions is similar to that after three consecutive abortions, suggesting that it is more reasonable to evaluate pregnancy loss after two consecutive spontaneous abortions. In women with pregnancy complications and older women it is very important to clarify the causes of spontaneous abortion as soon as possible to improve the success rate of subsequent pregnancy.^2^

The mechanism of RPL is complex, and in about half of cases the etiology of RPL is unknown. The common factors affecting RPL rates include heredity, autoimmune abnormalities, endocrine disorders, chromosome abnormalities, etc.^3^,^4^ The prethrombotic state has a certain impact on recurrent abortion. Hypercoagulation affects the blood flow state of the uterus and placenta, and may lead to the formation of local microthrombotic, or even placenta infarction, resulting in insufficient blood supply to the placenta, ischemia and hypoxia of the embryo or fetus, embryo- or fetal dysplasia and abortion.^5^ Thromboelastogram can identify the causes of abnormal coagulation function by monitoring the whole process of coagulation and fibrinolysis.^6^

Recent studies have suggested that more than 50% of recurrent abortions are related to immune disorder. However, the current research on this link is still limited. Additionally, there is still no consensus on the relationship between RPL and ACA, EmAb, ATA.^7^,^8^ The main goal of this study was to retrospectively explore the changes in coagulation indexes, thromboelastogram parameters and autoantibodies in RSA patients with different number of abortions to better understand a possible mechanism of RPL.

## METHODS

Medical records of the 48 patients with recurrent abortion that were treated in Quzhou people’s Hospital from November 2019 to October 2020 were collected as the observation group. Based on the number of abortions, they were divided into Group-A (two abortions, n=21), Group-B (three abortions, n=16) and group C (abortion≥4 times, n=11). The control group consisted of the records of 50 healthy pregnant women with no history of infertility and recurrent abortion, who were treated in the same period in our hospital, examined by routine autoantibody tests and thromboelastogram, and had a healthy baby.

### Inclusion criteria:

- Meet the RPL standard:^9^ Have spontaneous abortion twice or more with the same spouse, and the fetus is lost before 28 weeks of pregnancy;
- Time from last abortion ≥six months;
- Routine etiological examination results of recurrent abortion;
- There are routine results of autoantibodies and thromboelastogram.

### Exclusion criteria:

- Coagulation dysfunction alone or combined with immune system diseases;
- Chromosomal abnormalities in both men and women;
- Abnormal reproductive system structure;
- The history of heart disease, diabetes, hypertension, hemorrhagic diseases and thrombotic diseases;
- Combined with malignant tumor, major organic disease, and serious infection.

The age of the women in the observation-group was 21~35 years, with an average age of 29.68±2.76 years, and that of the control group was 25~35 years, with an average age of 29.50±2.58 years, with no statistical difference between the two groups (*P*>0.05). This study was approved by the medical ethics committee of our institution. (Dated. April 1^st^ 2020)

The following relevant indexes of pregnant women during pregnancy were collected:

- PT, APTT, FIB and DD. Briefly, 5ml of fasting venous blood was collected from pregnant women in the morning of the fourth week of pregnancy into sodium citrate vacuum anticoagulant tube, fully mixed, centrifuged at a rate of 3000r/minute for 10 minute to separate plasma. The coagulation indexes were detected by Beijing pulisheng automatic hemagglutination analyzer C2000-A.
- TEG parameters of thromboelastogram, including:^10^ R-the time from the initial detection to the formation of the first fibrin, with a normal range of 6~8 minutes; 2) K-the time required from the end of R time to the recording amplitude of TEG analyzer for 20 minutes (normal range is three to six minutes);
- MA, the maximum amplitude of TEG, which is mainly affected by platelet aggregation function and reflects the strength of forming blood clot, with a normal range of 50~60mm;
- Solidification angle(α), the tangent of the maximum curve radian of the tracing diagram from the end of R time. The included angle between this tangent and the horizontal line is the angle, and the normal range is 50~60. Beijing Lepu thromboelastography instrument CFMS LEPU-8800 was used to detect the TEG parameters of thromboelastography, and the operation steps were carried out according to the instructions.
- Levels of EmAb and ACA, measured by enzyme-linked immunosorbent assay (Shanghai Yuanye Biotechnology Co., Ltd) according to the manufacturer’s instructions.
- ATA was detected by electrochemiluminescence using Roche’s automatic electrochemical immunoassay system and supporting reagent detection kit. Anti-thyroglobulin antibody (TgAb) ≥ 4.11iu/ml, thyroid peroxidase antibody (TPOAb) ≥ 5.61ku/ml, are considered ATA positive.

The data were analyzed by SPSS 22.0 software. The count data were expressed in (%), Fisher exact probability method was used to test. The measurement data of normal distribution were represented by (*x̄*±*s*) using t-test. For one-way ANOVA, LSD-t test was used if the variance was homogeneous, and Dunnett-t test was used if the variance was not homogeneous. *P<*0.05 indicated that the difference was statistically significant.

## RESULTS

There were significant differences in the levels of ATTP, Pt, FIB and DD among the groups: higher number of abortions was associated with lower levels of ATPP and Pt, and higher the levels of FIB and DD, *P<*0.05, (Table-I).

**Table-I:** Comparison of coagulation function indexes in each group (*χ̅*±*S*).

Group	APTT (s)	PT (s)	FIB (g/L)	DD (ug/L)
Control group (n=50)	29.86±3.84	13.94±1.56	3.22±0.29	158.26±21.30
Group A (n=21)	26.62±3.56^*^	13.05±1.96^*^	3.86±0.56^*^	175.90±22.39^*^
Group B(n=16)	24.31±2.15^*a^	11.88±1.63^*a^	4.14±0.37^*a^	206.25±16.01^*a^
Group C(n=11)	21.45±2.34^* ab^	10.55±1.86^* ab^	4.47±0.49^* ab^	226.45±18.89^* ab^
F	24.404	15.149	45.754	45.942
P	<0.001	<0.001	<0.001	<0.001

***Note:***

* indicates that compared with the control group,

P<0.05;

a indicates that compared with Group-A,

P<0.05;

^b^ indicates that compared with Group-B, *P*<0.05.

Compared with the control group, R and K in Group A, B and C decreased, while solidification angle (α) and MA increased (p<0.05). There was no significant difference in R and K indexes between the groups A, B, and C (p>0.05), but there was significant difference in α and MA indexes between the three groups, *P<*0.05, (Table-II).

**Table-II:** Comparison of TEG parameters in each group (*χ̅*±*S*).

Group	R (min)	K (min)	α(*)	MA (mm)
Control-group (n=50)	3.64±0.99	1.16±0.31	78.98±1.66	65.40±3.74
Group A (n=21)	3.08±1.02^*^	0.87±0.23^*^	82.48±1.99^*^	71.05±3.90^*^
Group B(n=16)	2.89±0.66^*^	0.81±0.21^*^	85.37±2.68^*a^	74.25±3.96^*a^
Group C(n=11)	2.67±0.59^*^	0.76±0.18^*^	88.45±2.30^* ab^	78.45±6.33^* ab^
F	5.529	13.479	91.876	41.152
P	0.002	<0.001	<0.001	<0.001

***Note:***

* indicates that compared with the control group,

P<0.05;

a indicates that compared with Group-A,

P<0.05;

^b^ indicates that compared with Group-B, P<0.05.

The positive rates of ACA, EmAb and ATA in Group-A were higher than those in the control group, but these differences were not statistically significant (*P*>0.05), while the above indexes in groups B and C were significantly higher than those in the control group (*P*<0.05). The positive rates of ACA and ATA in group C were significantly higher than those in Group-A (*P*<0.05), while there was no significant difference in the positive rate of EmAb (*P*>0.05), *P<*0.05, Table-III.

**Table-III:** Comparison of positive rates of ACA, EmAb and ATA in each group (n, %).

Group	ACA positive	EmAb positive	ATA		

			ATA positive	TgAb positive	TPOAb positive
Control-group (n=50)	4(8%)	5(10%)	5(10%)	3(6%)	4(8%)
Group A (n=21)	5(23.81%)	6(28.57%)	6(28.57%)	3(14.29%)	6(28.57%)
Group B (n=16)	7(43.75%)^a^	8(50.00%)^a^	10(62.5%)^a^	4(19.05%)	7(43.75%)^a^
Group C (n=11)	8(72.73%)^a, b^	7(63.64%)^a^	9(81.82%)^a, b^	3(27.27%)	9(81.82%)^a, b^
Fisher	24.408	19.346	31.28	6.104	28.537
P	<0.001	<0.001	<0.001	0.059	<0.001

***Note:***

^*^ indicates that compared with the control group,

*P<*0.05;

a ^a^ indicates that compared with Group-A,

*P*<0.05;

b indicates that compared with Group-B, *P*<0.05.

## DISCUSSION

The results of our study showed that the coagulation indexes APTT and PT of RPL patients were significantly lower, while the levels of FIB and DD were significantly higher than those of healthy pregnant women (*P<*0.05). Higher number of abortions correlated with lower levels of APTT and PT, and higher levels of FIB and DD, suggesting that the risk of thrombosis in RPL patients was significantly higher than that in normal pregnant women. Studies show that increased levels of thrombosis increase the risk of RPL. Abnormal hemorheology, fibrinolysis, anticoagulation and coagulation of pregnant women during pregnancy may lead to pathological hypercoagulation, a prethrombotic state that, if continues to develop, will lead to thrombosis.^11^ Cavalcante MB *et al*^12^ showed that patients with RPL had a high risk of thromboembolic events. Studies have shown that patients with RPL often suffer from placental microthrombosis, which affects the normal placental blood circulation function and fails to provide normal blood oxygen for the fetus, resulting in fetal death.^13^ Zhang K *et al*^14^ pointed out that adequate uterine perfusion is very important for successful pregnancy. Since thrombosis easily leads to abnormal uterine perfusion, preventing thrombosis or improving uterine perfusion, therefore, can potentially improve pregnancy outcome and reduce the risk of abortion. Bao SH *et al*^15^ studied whether the plasma DD level could guide the anticoagulation treatment of RPL associated with antiphospholipid syndrome (APS), and confirmed that women with normal DD level had the highest live birth rate. The results of our study are consistent with these reports. At the same time, studies show that RPL patients have obvious overall coagulation dysfunction that is not related to pregnancy.^16^,^17^ Therefore, clinically, detecting the coagulation indexes of RPL patients and developing corresponding treatment plans are crucial to improve the success rate of pregnancy.

Thromboelastography (TEG) has been applied in many clinical fields. Branco BC *et al*^18^ used TEG to evaluate the effect of hypercoagulability in trauma patients. Shulutko EM *et al*^19^ evaluated the hemostatic effect of acetaminophen based on the readings of thromboelastogram and coagulation diagram. Gordon N *et al*^20^ evaluated perioperative TEG values to determine whether malignant tumor status affects blood coagulation after hepatectomy. Traditional coagulation test cannot show the process of coagulation. TEG, on the other hand, dynamically monitors the whole process of coagulation.^21^ The results of this study showed that there was no significant difference in the values of R and K among the three groups of women in the observation group. However, they were significantly lower in the observation group compared to the control group. At the same time, women in the observation group had significantly higher values of α and MA compared to the control group. The decrease of R and K and the increase of α and MA suggested that the patients in the observation group were in a hypercoagulable state.^18^,^19^ Therefore, TEG parameters can accurately reflect the difference between healthy pregnant women and RPL patients, and the risk of miscarriage in pregnant women with positive RPL history. The higher the number of abortions, the higher the values of α and MA, suggesting that the values of α and MA have certain guiding significance for the risk assessment of the number of recurrent abortions. Therefore, it is necessary to pay special attention to the values of α and MA in the TEG parameters of RPL patients in clinical practice.^19^ The α value reflects the rate of blood clot formation and reinforcement in vivo, while MA value reflects the function of platelet aggregation.^20^,^21^ Polokhov DM study^22^ pointed out that with the enhancement of platelet aggregation functions, patients’ coagulation function increases and fibrinolytic capacity decreases. This may easily lead to thrombosis in villous vessels and uterine spiral arteries in pregnant women, resulting in damage to vascular endothelium and embolism.

The results of our study show that the positive rates of ACA, EmAb and ATA in RPL patients with more than three abortions are significantly higher than those in the control group. This observation suggests that the high positive rates of ACA, EmAb and ATA may be a risk factors leading to multiple abortions in RPL patients, which is consistent with previous literature reports.^23^-^25^ EmAb is an organ specific antibody, while ACA and ATA are non-organ specific antibodies.^24^ Studies show that EmAb may react to endometrium as an antigen, and cause a series of immune responses.^23^ Antigen-antibody reaction with endometrium may interfere with the implantation and development of fertilized eggs, resulting in abortion.^24^,^25^ However, a prospective pilot case-control study on the relationship between serum EmAb and pregnancy and abortion by Parry *et al*^26^ showed that there was no significant correlation between EMAb and pregnancy and abortion. Therefore, the correlation between EmAb and recurrent abortion is still controversial. ATA includes TgAb and TPOAb. At present, the mechanism of abortion caused by ATA has not been determined, but some studies speculate that TgAb and TPOAb have a certain impact on embryo absorption and fetal immune system development, and can destroy the maternal balance during pregnancy, resulting in pathological pregnancy and even abortion.^27^,^28^ The potential mechanisms of ACA-induced abortion include developing placental circulation disorder that results in insufficient blood and oxygen supply to the fetus; placental vasculitis, resulting in insufficient fetal oxygen supply and nutrition; and thrombosis and vasoconstriction that reduce placental blood flow, and cause fetal distress and death.^29^,^30^ The results of this study also found that although the positive rates of the three antibodies in RPL patients with over four abortion were higher than those with over two abortions, only the positive rate indicators of ACA and ATA were statistically significant, while the positive rate indicators of EmAb were not statistically significant. This suggests that ACA and ATA, but not EmAb, are closely related to the number of abortions. At present, there is a lack of international research on the relationship between ACA, EmAb, ATA and specific abortion times, and further studies are needed to support our results.

### Limitations of the study

1) This is a single center study with a small sample size and relies on accurate, detailed, and available patient data due to its retrospective nature. In the future, the research design can be improved by expanding the sample population to support the conclusions of this study; Secondly, only ACA, EmAb, ATA and other auto antibody indicators are used. In the future, the relationship between auto antibody and RPL can be more comprehensively analyzed in combination with antisperm antibody (AsAb), antinuclear antibody (ANA) and other indicators; Thirdly the coagulation indexes such as APTT, PT, FIB, DD and TEG parameters are tested separately. In the future, the test results of the two can be combined to comprehensively evaluate the coagulation state after the action of various factors of abnormal coagulation and explore its related mechanism, to provide a scientific basis for the prevention and treatment of RPL in patients who may have hypercoagulable state.

## CONCLUSION

The mechanism of RPL is complex and is associated with the decrease in the levels of APTT and PT, and the increase in FIB and DD levels. TEG can better dynamically monitor the coagulation state of RPL patients. The increase of α angle and MA values correlates with the increased risk of multiple abortions. The increase in the positive rates of autoimmune antibodies ACA, EmAb and ATA is also closely related to multiple abortions, and the positive rates of ACA and ATA are closely related to the number of abortions. Therefore, paying attention to the coagulation indexes, TEG parameters and autoimmune antibody expression in patients with RPL may improve the prognosis in RPL patients.

### Authors’ contributions:

**HY:** Conceived and designed the study.

**HY & YJ & YZ:** Collected the data and performed the analysis.

**HY:** Was involved in the writing of the manuscript and is responsible for the integrity of the study.

All authors have read and approved the final manuscript.
